# Epidemiology and clinical characteristics of respiratory syncytial virus infections among children and adults in Mexico

**DOI:** 10.1111/irv.12414

**Published:** 2016-08-18

**Authors:** Ana E. Gamiño‐Arroyo, Sarbelio Moreno‐Espinosa, Beatriz Llamosas‐Gallardo, Ana A. Ortiz‐Hernández, M. Lourdes Guerrero, Arturo Galindo‐Fraga, Juan F. Galán‐Herrera, Francisco J. Prado‐Galbarro, John H. Beigel, Guillermo M. Ruiz‐Palacios, Daniel E. Noyola, the Mexico Emerging Infectious Diseases Clinical Research Network (La Red)

**Affiliations:** ^1^ Hospital Infantil de México Federico GómezMexico CityMexico; ^2^ Instituto Nacional de PediatríaMexico CityMexico; ^3^ Instituto Nacional de Ciencias Médicas y Nutrición Salvador ZubiránMexico CityMexico; ^4^ Mexico Emerging Infectious Diseases Clinical Research Network Coordinating CenterMexico CityMexico; ^5^ Agencia de Evaluación de Tecnologías SanitariasInstituto de Salud Carlos IIIMadridSpain; ^6^ Leidos Biomedical, Frederick, MD, in support of the National Institutes of Allergy and Infectious Diseases, National Institutes of HealthBethesdaMDUSA; ^7^ Universidad Autónoma de San Luis PotosíSan Luis PotosíMexico

**Keywords:** acute respiratory tract infections, bronchiolitis, influenza‐like illness, pneumonia, respiratory syncytial virus

## Abstract

**Background:**

Respiratory syncytial virus (RSV) is a leading etiological agent of acute respiratory tract infections and hospitalizations in children. However, little information is available regarding RSV infections in Latin American countries, particularly among adult patients.

**Objective:**

To describe the epidemiology of RSV infection and to analyze the factors associated with severe infections in children and adults in Mexico.

**Methods:**

Patients ≥1 month old, who presented with an influenza‐like illness (ILI) to six hospitals in Mexico, were eligible for participation in the study. Multiplex reverse‐transcriptase polymerase chain reaction identified viral pathogens in nasal swabs from 5629 episodes of ILI. Patients in whom RSV was detected were included in this report.

**Results:**

Respiratory syncytial virus was detected in 399 children and 171 adults. RSV A was detected in 413 cases and RSV B in 163, including six patients who had coinfection with both subtypes; 414 (72.6%) patients required hospital admission, including 96 (16.8%) patients that required admission to the intensive care unit. Coinfection with one or more respiratory pathogens other than RSV was detected in 159 cases. Young age (in children) and older age (in adults) as well as the presence of some underlying conditions were associated with more severe disease.

**Conclusions:**

This study confirms that RSV is an important respiratory pathogen in children in Mexico. In addition, a substantial number of cases in adults were also detected highlighting the relevance of this virus in all ages. It is important to identify subjects at high risk of complications who may benefit from current or future preventive interventions.

## 1 Introduction

Respiratory syncytial virus (RSV) is the major infectious cause of lower respiratory tract illness in infants and young children around the world.^1^, ^2^ It has also been recognized as an important etiologic agent of pneumonia and other respiratory tract infections in adults and elderly patients.^3^, ^4^ The clinical presentation of this infection varies widely, from mild upper respiratory tract disease to bronchiolitis and pneumonia.^5^ This virus is responsible for the majority of bronchiolitis cases and causes approximately 50% of pneumonia cases during the first years of life.^6^ In children, host factors such as young age, prematurity, and chronic cardiopulmonary diseases have been associated with severe disease. In addition, other factors such as lower socioeconomic status, exposure to cigarette smoke, air pollution, crowded households, and the lack of breastfeeding have also been associated with severe disease.^7^ Viral factors associated with virulence leading to severe disease are not sufficiently understood.^8^

Human RSV is a member of the *Paramyxoviridae* family. Outbreaks of RSV infections occur between fall and spring in temperate climates and tend to last up to 5 months.^9^, ^10^ RSV isolates can be divided into two groups: group A and group B based on antigenic and genetic characteristics.^8^ These two groups cocirculate in the human population, with group A being more prevalent. Several studies have compared the severity of disease between infants infected with RSV group A and group B with mixed results. Most studies have not found significant clinical differences between both subtypes.^8^ However, a possible effect of different viral strains on disease severity remains an open question.

Despite the recognized importance of RSV as a cause of respiratory illness, the information regarding the epidemiology of this virus in Latin America, particularly among adults, is limited.^11^ In the present study, 570 cases of RSV infection identified during four epidemic years in Mexico were evaluated to clarify the epidemiology of this infection and to assess the possible variations in demographic and clinical characteristics according to viral groups.

## 2 Materials and Methods

### 2.1 Study population

In 2009, a new influenza strain, influenza A(H1N1)pdm09 virus, was identified. This virus spread worldwide causing significant morbidity and mortality. It is recognized that multiple pathogens may present with respiratory symptoms, including influenza‐like illness (ILI). To improve our understanding of the epidemiological features of respiratory tract infections caused by influenza and other viruses in Mexico, a cohort study to determine the etiology and clinical characteristics of patients presenting with ILI was performed between 2010 and 2014. The study was conducted by the Mexican Emerging Infectious Diseases Clinical Research Network (La Red), a collaboration established by the Mexican Ministry of Health and the US National Institute of Allergy and Infectious Diseases. The study (ILI‐002 Study) was carried out at five hospitals in Mexico City and one in San Luis Potosí. Results from patients enrolled during the first year of the study have previously been described.^6^ In this report, we analyzed the characteristics of 570 patients (399 children and 171 adults) with confirmed RSV infection included in the study during a 4‐year period.

### 2.2 Case definition and selection criteria

Patients ≥1 month old who presented with an ILI to any of the participating hospitals were eligible for participation in the study. ILI was defined by the presence of at least one respiratory symptom (e.g., shortness of breath, nasal congestion, and cough) and one of the two following criteria: (i) fever ≥38°C on examination, or self‐reported fever, or feverishness in the past 24 hours; (ii) one or more non‐respiratory symptoms (e.g., malaise, headache, myalgia, or chest pain). In order to rule out a nosocomial infection, patients who had been hospitalized for more than 48 hours at the time of symptom onset were excluded from the study.

### 2.3 Study procedures

Subjects were interviewed and examined at the time of enrollment, a nasopharyngeal swab was obtained for the detection of respiratory pathogens, and a blood sample for the complete blood counts and chemistry analysis were obtained. When available, the results of other tests obtained for standard clinical care were extracted from medical records. Subjects were evaluated again on day 28 after enrollment, and follow‐up information was also obtained by a telephone call on day 14 after the enrollment. At each follow‐up visit, clinical information (symptoms, rehospitalizations, and death) was assessed. These visits allowed the ascertainment of the final disposition of the study patients (outpatient, emergency room, hospital ward, or intensive care unit [ICU]).

### 2.4 Laboratory diagnostics

Nasopharyngeal swab specimens were collected from enrolled patients, placed in a tube with viral transport media, and maintained under refrigeration until they were sent to the Molecular Biology Laboratory of the Infectious Diseases Department, Instituto Nacional de Ciencias Médicas Salvador Zubirán, where they were stored at −70°C. Samples from the patients enrolled in San Luis Potosí were maintained under refrigeration and transported to the Medical School (Universidad Autónoma de San Luis Potosí) where they were stored at −70°C until they were sent to the Instituto Nacional de Ciencias Médicas Salvador Zubirán for storage and virological testing; the samples sent from San Luis Potosí to Mexico City were shipped on dry ice.

All nasopharyngeal swabs were tested by real‐time polymerase chain reaction (RT‐PCR) for influenza A following the Centers for Disease Control and Prevention protocol as described previously.^12^ Respiratory samples were also tested with the RespiFinder 19 (April 2010 to May 2012) or RespiFinder 22 (previously RespiFinder Plus, June 2012 to March 2014), from PathoFinder BV, Maastricht, the Netherlands. This multiplex RT‐PCR test can detect and differentiate 15 viruses (coronavirus NL63, OC43, and 229E, human metapneumovirus, influenza A, influenza AH5N1, influenza B, parainfluenza virus types 1 to 4, RSV A and B, rhinovirus, and adenovirus), as well as four bacteria (*Bordetella pertussis*,* Chlamydophila pneumoniae*,* Legionella pneumophila*, and *Mycoplasma pneumoniae*). RespiFinder 22 removed influenza H5N1 and added bocavirus (type 1), coronavirus HKU1, influenza A H1N1v, and enterovirus.

### 2.5 Ethical considerations

Verbal consent was obtained from parents/legal tutor and subjects before screening to determine eligibility. Once a patient was determined to be eligible for study participation, written informed consent and assent (for children older than 8 years) were obtained. The protocol was approved by the institutional review board of each hospital.

### 2.6 Statistical analysis

Means and standard deviation were used to summarize the quantitative variables, while frequencies and percentages were used for the qualitative variables. Comparisons between groups were made using Student's *t*‐test for quantitative variables and the chi‐squared or Fisher's exact test for qualitative variables. Multivariate logistic regression analysis was used to determine the factors associated with hospitalization and ICU admission. Data were analyzed with the use of pspp and OpenEpi. A *P* value <.05 was considered as statistically significant.

## 3 Results

**Figure 1:**
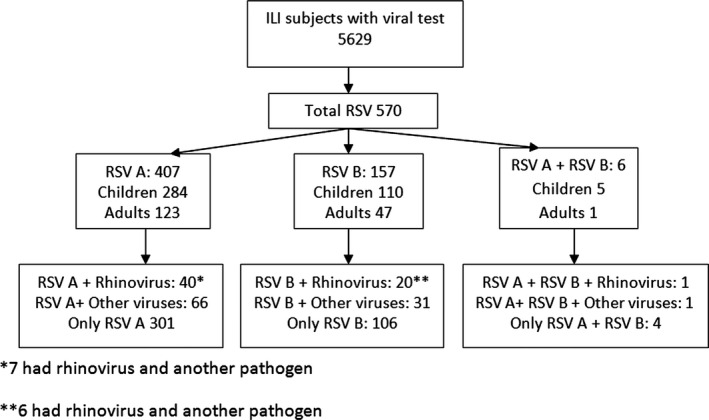
Respiratory syncytial virus infections in subjects included during four seasons in the ILI‐002 study

Between 2010 and 2014, there were 5662 subjects enrolled in the ILI‐002 study; 33 patients did not have respiratory samples available for viral testing, and therefore, the final sample size for analysis was 5629 subjects. Of these subjects, there were 570 (10.7%) cases with RSV infection: 407 with RSV A infection, 157 with RSV B, and six in whom both RSV A and B were detected (Fig. 1).

**Figure 2:**
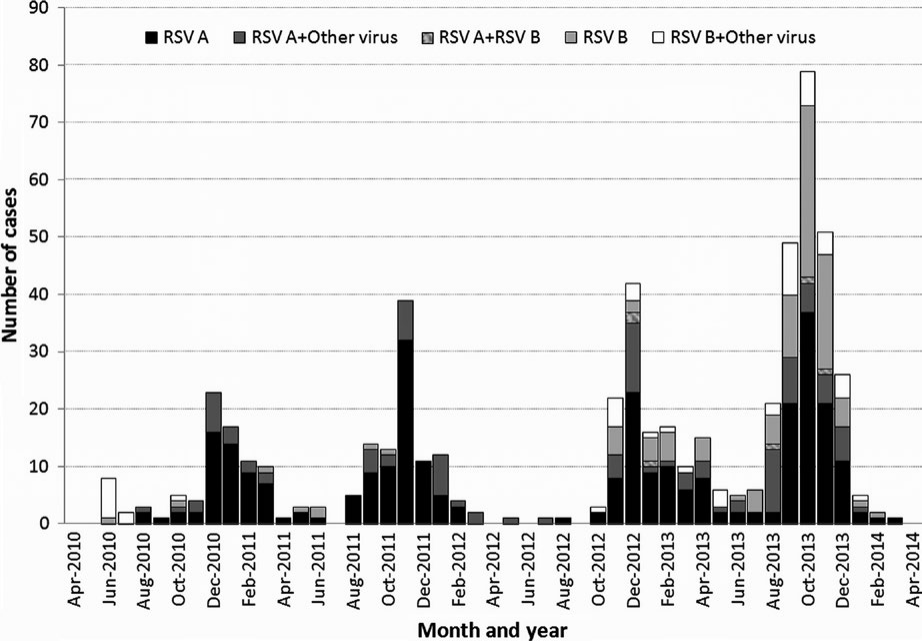
Respiratory syncytial virus seasonality according to the viral subtype and presence of coinfection

RSV cases were detected throughout the study period. However, a marked seasonality was observed with a high number of cases during the fall and winter and a decrease in detections during spring and summer (Fig. 2). RSV A was the predominant virus between 2010 and 2012, but in seasons between 2012 and 2014, both RSV subtypes circulated simultaneously.

The characteristics of patients with RSV infection were compared to those of all other patients enrolled in the ILI‐002 study (Table 1). The age group affected more frequently by RSV was that of children 0–5 years of age; 66.84% of RSV infections occurred in children in this age group, while 30% occurred in adults and 3.16% in children 6–17 years of age; 24.6% (381/1550) of respiratory tract infections in children <5 years were caused by RSV as compared to 4.7% (18/379) of children older than 5 years and 4.6% (171/3700) of adults. Also, the proportion of cases seen as outpatients was lower in patients with RSV infection compared to those in which this virus was not detected, while the proportion of ICU admissions was higher (Table 1).

**Table 1:** Characteristics of patients with ILI and RSV infections compared to those in whom respiratory syncytial virus was not detected

	RSV(n=570)	RSV negative(n=5059)	*P*
Final disposition			
Outpatient	156 (27.4%)	2700 (53.4%)	<.001
Hospitalized^a^	318 (55.8%)	1979 (39.1%)	
Intensive care unit	96 (16.8%)	380 (7.5%)	
Gender			
Female	272 (47.7%)	2954 (58.4%)	<.001
Male	298 (52.3%)	2105 (41.6%)	
Age group			
0–5 y	381 (66.8%)	1169 (23.1%)	<.001
6–17 y	18 (3.1%)	361 (7.1%)	
18–49 y	108 (18.9%)	2572 (50.8%)	
>50 y	63 (11%)	957 (18.9%)	

a Includes patients in emergency room and hospital wards.

**Figure 3:**
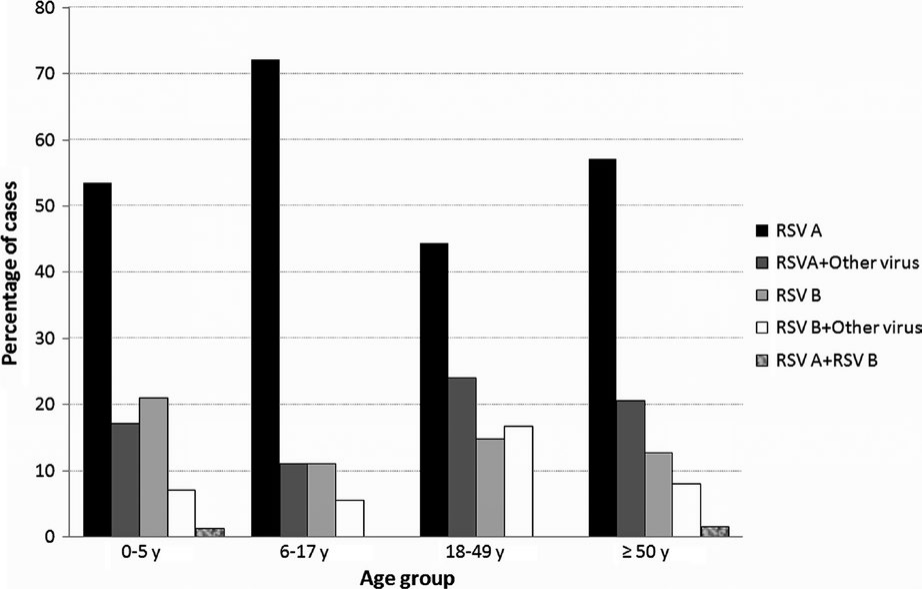
Proportion of respiratory syncytial virus viral subtype and the presence of coinfection according to age group

In 159 (28%) patients, another pathogen was detected in addition to RSV. In 136 of these patients, there was one additional pathogen detected, while in 22 cases there were two, and in one case, there were three; these included rhinovirus (n=61), coronaviruses (n=30), parainfluenza viruses (n=17), influenza A (n=17), influenza B (n=12), adenovirus (n=12), metapneumovirus (n=11), *Bordetella pertussis* (n=11), *Mycoplasma* (n=6), and bocavirus (n=6). The proportions of infections caused by each RSV subtype and the presence of coinfections, according to the age group, are shown in Fig. 3.

We compared the characteristics of infections caused by RSV A and RSV B to assess whether there were significant clinical differences between them (Table 2); the six cases with coinfection with RSV A and B were excluded from this analysis. We did not find any differences in the medical history between patients with RSV A and RSV B infections. We also compared the frequency of each symptom between patients with RSV A and RSV B infections; no significant differences were observed for any of the symptoms that were recorded (including fever, cough, sore throat, fatigue, headache, myalgias, eye symptoms, sneezing, rhinorrhea, shortness of breath, nausea, vomiting, diarrhea, confusion, malaise, and irritability) (data not shown).

**Table 2:** Demographic and clinical characteristics of patients with RSV A and RSV B infections

Characteristics	RSV A^a^ (n=407)	RSV B^a^(n=157)	*P*
Age			
0–5 y	269 (66.1%)	107 (68.1%)	0.34
6–17 y	15 (3.7%)	3 (1.9%)	
18–49 y	74 (18.2%)	34 (21.7%)	
>50 y	49 (12%)	13 (8.3%)	
Gender			
Male	207 (50.9%)	88 (56%)	0.27
Female	200 (49.1%)	69 (43.9%)	
Smoking history			
Never smoked	299 (73.5%)	114 (72.6%)	0.84
Former/current/passive smoker	108 (26.5%)	43 (27.4%)	
Underlying disorders			
Any underlying disorder	173 (42.5%)	68 (43.3%)	0.86
Cardiovascular disorder	32 (7.9%)	10 (6.4%)	0.54
Impairment of activities of daily living	2 (0.5%)	2 (1.3%)	0.31
Hematological malignancy	4 (0.9%)	0	0.58
Chronic lung disease	13 (3.2%)	7 (4.5%)	0.47
Solid organ malignancy	2 (0.5%)	0	1
Neuromyopathy	1 (0.2%)	0	1
Diabetes mellitus	3 (0.7%)	2 (1.3%)	0.62
Immunodeficiencies	6 (1.5%)	3 (1.9%)	0.71
Congenital malformations/congenital syndromes	49 (12%)	25 (15.9%)	0.22
Renal disorder	1 (0.2%)	1 (0.6%)	0.48
HIV infection	2 (0.5%)	1 (0.6%)	1
Asthma	28 (6.9%)	8 (5.1%)	0.56
Cognitive dysfunction	4 (0.9%)	7 (4.5%)	0.013
Site of medical attention			
Hospitalized	294 (72.2%)	115 (73.2%)	0.81
ICU admission	60 (14.7%)	33 (21%)	0.072

a Does not include six cases of RSV A+RSV B coinfection.

In addition, we compared the clinical characteristics of patients with and without coinfection with other pathogens (Table 3). Differences in the age group distribution were noted between cases with and without coinfection with other pathogens: The proportion of pediatric cases was higher for those infections caused by RSV only, while a larger proportion of coinfections was noted in adults. Most symptoms were as frequent in patients with infections caused by RSV only compared to those with coinfections (data not shown); however, there were differences in the frequencies of some symptoms: Patients with coinfections reported fatigue (51.6% vs 36.9%; *P*=.001) and headache (28.3% vs 19.5%; *P*=.02) more frequently than those without coinfections; on the other hand, the presence of rales (24.5% vs 33.3%; *P*=.04), wheezing (50.9% vs 61.1%; *P*=.03), and shortness of breath (61% vs 70.1%; *P*=.04) was noted less frequently among patients with coinfections compared to those in whom RSV was the only pathogen. The proportion of hospitalized patients was higher in those infected by RSV only compared to those with other respiratory pathogens (77.1% vs 61%; *P*<.001); however, no increase in the proportion of ICU admissions was noted (16.5% vs 17.6%; *P*=.76).

**Table 3:** Demographic and clinical characteristics of patients with respiratory syncytial virus and coinfection with other respiratory pathogens

Characteristics	RSV only(n=411)	RSV and other respiratory pathogens(n=159)	*P*
Age			
0–5 y	287 (69.8%)	94 (59.1%)	0.007
6–17 y	15 (3.6%)	3 (1.9%)	
18–49 y	64 (15.6%)	44 (27.7%)	
>50 y	45 (10.9%)	18 (11.3%)	
Gender			
Male	217 (52.8%)	81 (50.9%)	0.69
Female	194 (47.2%)	78 (49.1%)	
Smoking history			
Never smoked	315 (76.6%)	101 (63.5%)	0.002
Former/current/passive smoker	96 (23.4%)	58 (36.5%)	
Underlying disorders			
Any underlying disorder	181 (44%)	61 (38.4%)	0.22
Cardiovascular disorder	30 (7.3%)	13 (8.2%)	0.72
Impairment of activities of daily living	1 (0.2%)	3 (1.9%)	0.068
Hematological malignancy	2 (0.5%)	2 (1.3%)	0.31
Chronic lung disease	19 (4.6%)	1 (0.6%)	0.02
Solid organ malignancy	1 (0.2%)	1 (0.6%)	0.48
Neuromyopathy	1 (0.2%)	0	1
Diabetes mellitus	4 (0.9%)	1 (0.6%)	1
Immunodeficiencies	6 (1.5%)	3 (1.9%)	0.71
Congenital malformations/congenital syndromes	57 (13.9%)	17 (10.7%)	0.31
Renal disorder	1 (0.2%)	1 (0.6%)	0.48
HIV infection	2 (0.5%)	1 (0.6%)	1
Asthma	27 (6.6%)	9 (5.7%)	0.69
Cognitive dysfunction	8 (1.9%)	3 (1.9%)	1
Site of medical attention			
Hospitalized	317 (77.1%)	97 (61%)	<0.001
ICU admission	68 (16.5%)	28 (17.6%)	0.76

To further assess the role of coinfections and other factors in the severity of RSV infections, we compared the characteristics of patients admitted to the hospital and those that were treated only as outpatients. Because of the differences in risk factors described in pediatric and adult patients, a separate analysis was carried out for these two groups (Tables 4 and 5). Variables with a significant association on univariate analysis (*P*<.05) were included in a multivariate logistic regression model. In children, factors with a significant independent association with RSV hospitalization included patient's age (younger children with higher risk) and the presence of another respiratory pathogen (reduced risk in those with coinfection) (Table 6). In adults, there was a significant association between patient's age (older patients with higher risk; odds ratio (OR) 1.06; 95% CI, 1.03–1.09, *P*= <.001), sex (higher risk in males; OR 0.26; 95% CI, 0.11–0.62, *P*= .002), the presence of any underlying conditions (OR 2.56; 95% CI, 1.01–6.51, *P*=.048), and asthma (OR 10.93; 95% CI, 2.53–47.24, *P*=.001) and the risk of hospitalization (Table 6).

**Table 4:** Factors associated with hospitalization in patients with respiratory syncytial virus infection in pediatric patients (<18 years of age)

Characteristics	Outpatient(n=55)	Hospitalized(n=344)	*P* value
RSV A	42 (76.4%)	242 (70.3%)	0.36
RSV B	12 (21.8%)	98 (28.5%)	0.31
RSV A and B coinfection	1 (1.8%)	4 (1.2%)	>0.99
Other respiratory pathogen	23 (41.8%)	74 (21.5%)	0.001
Age (years) (mean, SD)	2.4 (3.2)	1.3 (2.1)	0.018
Gender			
Male	29 (52.7%)	204 (59.3%)	0.36
Female	26 (47.3%)	140 (40.7%)	
Smoking history			
Never smoked	45 (81.8%)	277 (80.5%)	0.82
Former/current/passive smoker	10 (18.2%)	67 (19.5%)	
Underlying conditions			
Any underlying condition	14 (25.4%)	142 (41.3%)	0.026
Cardiovascular disorder	0	8 (2.3%)	0.61
Activities of daily living impaired	0	2 (0.6%)	1
Hematological malignancy	0	3 (0.9%)	1
Chronic lung disease	2 (3.6%)	12 (3.5%)	1
Solid organ malignancy	0	1 (0.3%)	1
Neuromyopathy	0	1 (0.3%)	1
Immunodeficiencies	1 (1.8%)	4 (1.2%)	0.53
Congenital malformations/congenital syndromes	4 (7.3%)	68 (19.8%)	0.023
Renal disorder	0	1 (0.3%)	1
Asthma	2 (3.6%)	14 (4.1%)	1
Cognitive dysfunction	2 (3.6%)	9 (2.6%)	0.65

**Table 5:** Factors associated with hospitalization in patients with respiratory syncytial virus infection in adult patients (≥18 years of age)

Characteristics	Outpatient(n=101)	Hospitalized(n=70)	*P* value
RSV A	71 (70.3%)	52 (74.3%)	0.57
RSV B	30 (29.7%)	17 (24.3%)	0.43
RSV A and B coinfection	0	1 (1.4%)	0.82
Other respiratory pathogen	39 (38.6%)	23 (32.9%)	0.44
Age (years) (mean, SD)	36.5 (14.6)	55.4 (18.1)	<0.001
Gender			
Male	30 (29.7%)	35 (50%)	0.007
Female	71 (70.3%)	35 (50%)	
Smoking history			
Never smoked	61 (60.4%)	33 (47.1%)	0.087
Former/current/passive smoker	40 (39.6%)	37 (52.9%)	
Underlying conditions			
Any underlying condition	29 (28.7%)	57 (81.4%)	<0.001
Cardiovascular disorder	16 (15.8%)	19 (27.1%)	0.072
Activities of daily living impaired	0	2 (2.9%)	0.17
Hematological malignancy	0	1 (1.4%)	0.41
Chronic lung disease	0	6 (8.6%)	0.004
Solid organ malignancy	1 (0.9%)	0	1
Diabetes mellitus	1 (0.9%)	4 (5.7%)	0.16
Immunodeficiencies	3 (2.9%)	1 (1.4%)	0.64
Congenital malformations/congenital syndromes	1 (0.9%)	1 (1.4%)	1
Renal disorder	0	1 (1.4%)	0.41
HIV infection	3 (2.9%)	0	0.27
Asthma	3 (2.9%)	17 (24.3%)	<0.001

**Table 6:** Multivariate logistic regression analysis of factors associated with hospitalization in patients with respiratory syncytial virus infection

Characteristics	OR (95% CI)	Adjusted *P* value
Pediatric cases		
Other respiratory pathogen	0.38 (0.21–0.7)	.002^a^
Age (years)	0.83 (0.74–0.92)	.001^a^
Any underlying condition	2.03 (0.9–4.56)	.086
Congenital malformations/congenital syndromes	2.01 (0.58–6.96)	.273
Adult cases		
Age (years)	1.06 (1.03–1.09)	<.001^a^
Gender		
Male	1	
Female	0.26 (0.11–0.62)	.002^a^
Any underlying condition	2.56 (1.01–6.51)	.048^a^
Chronic lung disease	NA	.99
Asthma	10.93 (2.53–47.24)	.001^a^

a Statistically significant.

Finally, we assessed the factors associated with admission to the ICU in hospitalized patients. Only three of the 96 patients that required ICU admission were adults; therefore, analysis of associated factors was limited to the group of children (Table 7). Children with RSV A infections were admitted less frequently to the ICU than those with RSV B infection; however, after multivariate analysis, this difference was not statistically significant (*P*=.227). Detection of other respiratory pathogens in addition to RSV (OR 1.9; 95% CI, 1.08–3.36, *P*=.027), as well as the presence of cardiovascular disorder (OR 22.36; 95% CI, 2.67–187.44, *P*= .004) and chronic lung disease (OR 4.67; 95% CI, 1.42–15.39, *P*= .011), showed a significant association with the need of admission to the ICU in this analysis limited only to children.

**Table 7:** Factors associated with ICU admission in children hospitalized with respiratory syncytial virus infection

Characteristics	Hospitalized without ICU(n=251)	ICU(n=93)	*P* value
RSV A	184 (73.3%)	58 (62.4%)	0.048
RSV B	66 (26.3%)	32 (34.4%)	0.14
Coinfection RSV	1 (0.4%)	3 (3.2%)	0.12
Other respiratory pathogen	47 (18.7%)	27 (29%)	0.039
Age (years)(mean, SD)	1.4 (2)	1.1 (2.2)	0.23
Gender			
Male	146 (58.2%)	58 (62.4%)	0.48
Female	105 (41.8%)	35 (37.6%)	
Smoking history			
Never smoked	207 (82.5%)	70 (75.3%)	0.13
Former/current/passive smoker	44 (17.5%)	23 (24.7%)	
Underlying conditions			
Any underlying condition	100 (39.8%)	42 (45.2%)	0.37
Cardiovascular disorder	1 (0.4%)	7 (7.5%)	0.001
Impairment of activities of daily living	0	2 (2.1%)	0.073
Hematological malignancy	2 (0.8%)	1 (1.1%)	1
Chronic lung disease	5 (1.9%)	7 (7.5%)	0.013
Solid organ malignancy	1 (0.4%)	0	1
Neuromyopathy	0	1 (1.1%)	0.27
Immunodeficiencies	4 (1.6%)	0	0.58
Congenital malformations/congenital syndromes	48 (18.1%)	20 (21.5%)	0.62
Renal disorder	1 (0.4%)	0	1
Asthma	12 (4.8%)	2 (2.1%)	0.37
Cognitive dysfunction	9 (3.6%)	0	0.12

ICU, intensive care unit.

## 4 Discussion

We present the characteristics of RSV infections detected in a prospective, multicenter study of children and adults seeking clinical care. Our data obtained over four seasons provide a description of the epidemiology of RSV infection in Mexico among people of all ages that presented to hospital with ILI. Infections caused by this virus were detected year‐round, but had a notable seasonal distribution with high circulation during the fall and winter. Overall, the highest numbers of detections were recorded between October and December. The predominant subtype was RSV A over the four seasons, although during the last season included in the study the number of RSV B detections was similar to that of RSV A.

In recent years, there has been a growing interest on the impact of RSV infection in adults. RSV has been reported to be one of the leading causes of community‐acquired pneumonia in adults, just behind *Streptococcus pneumonia* and influenza virus.^3^ In this study, RSV was detected in 4.6% of adults with respiratory tract infections and ILI. Previous studies have also found a significant role of RSV in adults; for instance, Johnstone et al.^13^detected the presence of RSV in 2.6% of cases of community‐acquired pneumonia in adults. In a recent study including adults with respiratory tract infections assessed in the emergency room or in the hospital, RSV was also detected in 2.6% of patients.^14^ In another study, RSV was detected in 7.4% of respiratory illnesses in subjects ≥ 65 years from 14 countries with moderate‐to‐severe ILI and was more prevalent in hospitalized patients.^15^ Thus, our results are consistent with previous reports suggesting that RSV is an important cause of moderate‐to‐severe ILI and may be associated with hospitalization in the elderly.^16^

In this study, multiple viruses were detected in some samples from children and adults because of the use of a multiplex RT‐PCR assay, which can detect 19–22 different respiratory pathogens. The combination of RSV A and rhinovirus was the coinfection most frequently found. The role of other viruses during RSV infection has been subject of previous studies.^17^ Rhinovirus is one of the most frequently reported viruses in combination with RSV.^18^ An increase in disease severity during viral coinfection has been suggested in several of these reports. However, not all studies concur in their results.^19^ In our study, we found the differences in this regard between children and adults; while no apparent effect of viral coinfections was observed in adults, children with RSV infection in whom other pathogens were detected were hospitalized less frequently than those infected by RSV only. However, among hospitalized children, the presence of another respiratory pathogen was associated with admission to the ICU. In order to interpret these results, it is important to take into account that there was a wide variety of coinfecting pathogens and that in some cases two and even three viruses, in addition to RSV, were detected.

One of the main objectives of the study was to identify the factors associated with severe disease leading to RSV hospitalization in our country. Approximately 40% of subjects with RSV infection that were enrolled in the study had at least one underlying disorder. The most frequent conditions included congenital malformations/congenital syndromes, cardiovascular disorders, chronic lung disease, and asthma. We observed significant differences in the factors associated with hospitalization between children and adults; while multivariate logistic regression analysis indicated that young age was the main variable associated with hospitalization in children, adults showed an association between additional factors and the requirement of hospitalization. In this age group, multivariate logistic regression analysis showed an association between RSV infection and age, the presence of any underlying condition, and asthma. In their global study of respiratory tract viral infections in elderly adults, Falsey et al.^15^found that congestive heart failure, other cardiopulmonary diseases, and noninflammatory cerebrovascular/neurological disorders appeared to be associated with RSV infection; however, on multivariate analysis, no specific factors were associated with this virus.

In the group of children, multivariate logistic regression analysis showed an association between the risk of ICU admission and the presence of coinfection with other respiratory pathogens, cardiovascular disorders, and chronic obstructive lung disease. In a cohort study of children with severe RSV infection conducted in England, multiple preexisting diseases (relative risk: 4.38) and congenital cardiac defects (relative risk: 2.98) were considered risk factors for death from severe RSV infection.^20^ Recently, in a multicenter study performed in Spain, Moreno‐Perez et al.^21^ compared the clinical characteristics of two groups of children with RSV infection: those “with” vs “without” underlying diseases. They found that patients with respiratory tract diseases required oxygen therapy more often (OR: 2.99; 95% CI, 1.03–8.65). In addition, mechanical ventilation was used more in patients with cardiac diseases (OR: 3.0; 95% CI, 1.07–8.44) and in those with inborn errors of metabolism (OR: 12.27; 95% CI, 2.11–71.47); this subgroup showed a higher risk of admission to the ICU (OR: 6.7, 95% CI, 1.18–38.04).^21^

In the present study, we have detected no significant difference in severity between subgroup A and subgroup B RSV infection. Some reports have indicated that subgroup A RSV was associated with more severe disease, whereas others have found the converse.^22^ In this regard, the systematic and prospective collection of detailed clinical data in our study, as well as the inclusion of patients of a wide spectrum of disease severity, supports the conclusion that no clinical significant differences in disease presentation are seen between the major RSV subtypes. Whether specific strains within each subgroup are associated with the severity of illness remains unclear. For instance, Martinello et al.^23^ showed that clade GA3 was associated with greater severity of illness. However, strains belonging to this clade appear to be a minority among currently circulating RSV strains.^24^

Our study is subject to some limitations. To be eligible for study participation, subjects had to fulfill the ILI definition established in this project. As such, not all patients with respiratory tract infections were eligible for inclusion. Nevertheless, our definition of ILI allowed for a wide spectrum of disease severity to be included in the study. Other limitations include the fact that the presence of underlying conditions (including history of prematurity and other chronic diseases) was based on patient self‐report, which might not be totally reliable. Also, we did not collect data on palivizumab use which might be relevant in infants; however, access to this prophylaxis is limited in Mexico because of the high cost and, therefore, the number of patients that may have received it is likely to be low.

On the other hand, strengths of our study include the participation of several institutions and the inclusion of patients of all ages. This allows us to report the characteristics of RSV infection in children and adults in Mexico. Our study illustrates the importance of RSV as a cause of serious respiratory illness affecting not only pediatric patients. We observed that, similar to influenza, young infants and the elderly show higher proportions of RSV‐associated hospitalizations than middle‐aged adults. However, the precise role of different underlying conditions as risk factors for severe infection in this population could not be established. Additional prospective studies are needed to identify populations that may benefit from current or future preventive interventions against RSV, such as vaccines.

## Author Contributions

Ana E. Gamiño‐Arroyo (Hospital Infantil de México Federico Gomez) involved in concept and design, data analysis and correlation of results, writing of manuscript, and final approval of version to be published. Sarbelio Moreno‐Espinosa (Hospital Infantil de México Federico Gomez) involved in coordinating patient enrollment and data collection, interpretation of results, revision of intellectual content of the manuscript, and final approval of version to be published. Beatriz Llamosas‐Gallardo (Instituto Nacional de Pediatría) involved in coordinating patient enrollment and data collection, interpretation of results, revision of intellectual content of the manuscript, and final approval of version to be published. Ana A. Ortíz‐Hernández (Instituto Nacional de Pediatría) involved in project coordination, patient enrollment and data collection, interpretation of results, revision of intellectual content of the manuscript, and final approval of version to be published. M. Lourdes Guerrero‐Almeida (Instituto Nacional de Ciencias Médicas y Nutrición Salvador Zubirán) involved in data collection, database revision, interpretation of results, revision of intellectual content of the manuscript, and final approval of version to be published. Arturo Galindo‐Fraga (Instituto Nacional de Ciencias Médicas y Nutrición Salvador Zubirán) involved in project coordination, patient enrollment and data collection, interpretation of data and results, revision of intellectual content of the manuscript, and final approval of version to be published. Juan F. Galán‐Herrera involved in coordination of the various hospitals in the network, coordination of data and sample collection, revision of intellectual content of the manuscript, and final approval of version to be published. Francisco J. Prado‐Galbarro involved in statistical analysis, interpretation of data, revision of intellectual content of the manuscript, and final approval of version to be published. John H. Beigel (Leidos Biomedical) involved in project coordination, interpretation of data and results, revision of intellectual content of the manuscript, and final approval of version to be published. Guillermo M. Ruiz‐Palacios (Instituto Nacional de Ciencias Médicas y Nutrición Salvador Zubirán) involved in project coordination, interpretation of data and results, revision of intellectual content of the manuscript, and final approval of version to be published. Daniel E. Noyola (Universidad Autónoma de San Luis Potosí) involved in concept and design, data analysis and correlation of results, writing of manuscript, and final approval of version to be published.

## Funding

La Red is funded by the Mexico Ministry of Health and the U.S. National Institute of Allergy and Infectious Diseases. This project has been funded in part by funding provided by CONACYT (Fondo Sectorial SSA/IMSS/ISSSTE, Projects No. 71260 and No. 127088); National Institute of Allergy and Infectious Diseases, National Institutes of Health, through its Intramural Research Programs and a contract with Westat, Inc., Contract Number: HHSN2722009000031, Task Order Number: HHSN27200002; and through the National Cancer Institute, National Institutes of Health, under Contract No. HHSN261200800001E. The content of this publication does not necessarily reflect the views or policies of the Department of Health and Human Services, or Westat, nor does mention of trade names, commercial products, or organizations imply endorsement by the US Government.

## Competing Interest Statement

D. E. Noyola has participated as a member of the speakers' bureau of AbbVie. The rest of the authors declare that they have no relevant conflicts of interest to report.
